# A Hyphenated Preconcentrator-Infrared-Hollow-Waveguide Sensor System for N_2_O Sensing

**DOI:** 10.1038/s41598-018-23961-8

**Published:** 2018-04-12

**Authors:** João Flavio da Silveira Petruci, Andreas Wilk, Arnaldo Alves Cardoso, Boris Mizaikoff

**Affiliations:** 1 São Paulo State University, Department of Analytical Chemistry, UNESP, CEP 14800-970 Araraquara, SP Brazil; 2 Ulm University, Institute of Analytical and Bioanalytical Chemistry, 89081 Ulm, Germany

## Abstract

Following the Kyoto protocol, all signatory countries must provide an annual inventory of greenhouse-gas emission including N_2_O. This fact associated with the wide variety of sources for N_2_O emissions requires appropriate sensor technologies facilitating *in-situ* monitoring, compact dimensions, ease of operation, and sufficient sensitivity for addressing such emission scenarios. In this contribution, we therefore describe an innovative portable mid-infrared chemical sensor system for quantifying gaseous N_2_O via coupling a substrate-integrated hollow waveguide (iHWG) simultaneously serving as highly miniaturized mid-infrared photon conduit and gas cell to a custom-made preconcentrator. N_2_O was collected onto a solid sorbent material packed into the preconcentrator unit, and then released via thermal desorption into the iHWG-MIR sensor utilizing a compact Fourier transform infrared (FTIR) spectrometer for molecularly selective spectroscopic detection with a limit of detection (LOD) at 5 ppbv. Highlighting the device flexibility in terms of sampling time, flow-rate, and iHWG design facilitates tailoring the developed preconcentrator-iHWG device towards a wide variety of application scenarios ranging from soil and aquatic emission monitoring and drone- or unmanned aerial vehicle (UAV)-mounted monitoring systems to clinical/medical analysis scenarios.

## Introduction

Nitrogen was first identified as an essential nutrient for all plants and humans in the middle of the 19^th^ century^1,2^. Since the remarkable invention of the Haber-Bosch process in the beginning of the last century, an unlimited supply of Nr has been deposited worldwide, thus enabling an abrupt growth of the human population due the increase in food availability^3^.

Approximately 100 years after the introduction of the Haber-Bosch process, a study led by Rockstrom and colleagues^4^ proposed a safe operating space for mankind, is associates the earth as an eco-system with anthropogenic procedures and their thresholds. If certain boundaries are crossed, unacceptable environmental changes may be initiated putting the rather delicate environmental balance at risk. Among the procedures that have already extensively exceeded the safety threshold is the nitrogen cycle. As mentioned, large amounts of reactive nitrogen are annually applied into the soil during food production as a major component of fertilizers. Recent studies have shown that less then 40% of Nr is used by plants, thus resulting in an excess of reactive nitrogen spreading into various environmental compartments^3^. Most nitrogen-based fertilizers use soluble nitrates, urea or ammonium. Consequently, lixiviation of soluble Nr to aquatic systems such as rivers, lakes, estuaries, and aquifers has been described of major environmental concern^5^. This fact results in thousands of tons of Nr annually moved to atmosphere as volatile forms (NH_3_, NOx and N_2_O), which contributes to extend the range of this pollution to regional and even global scale^6,7^.

According to the nitrogen cycle, both bacterial nitrification and denitrification pathways are responsible for the emission of nitrous oxide into the atmosphere^8^. Since the introduction of the Haber-Bosch process, the background levels of N_2_O in atmosphere have increased by almost 20% from 270 to 330 ppb^8^. Nitrous oxide is considered a major greenhouse gas next to carbon dioxide and methane with an estimated contribution of approx. 6% to global warming, despite the fact that N_2_O is present at much lower concentration than CO_2_. The global warming potential (GWP) of N_2_O is approximately three hundred times larger than CO_2_. Besides, in the stratosphere N_2_O is oxidized to NO and NO_2_, which are both among the constituents responsible for the destruction of the ozone layer. Hence, next to its role as environmental contaminant, nitrous oxide is considered among the most relevant ozone-depleting gas during in the 20^th^ century^9^.

According to the Kyoto protocol, all signatory nations must provide an annual greenhouse gas inventory, which should include N_2_O^10^. However, establishing the N_2_O budget is non-trivial due to (i) the low concentration of N_2_O in the atmosphere (i.e., in the ppbv range) when compared to CO_2_, and (ii) the spatial and temporal variations in the episodes of N_2_O emission from soil and aquatic systems^9^. In addition, a wide range of factors affects the rate of emission including but not limited to pH, temperature, moisture, soil composition, and microflora^5^. Currently, extensive research has been focused on fundamentally understanding the processes involved in N_2_O emissions from soil and aquatic systems, and have triggered substantial interest in the to the development of analytical strategies and methods providing reliable and accurate data^11–15^.

Latent emissions provided by aquatic systems and soil reactions are believed to be the dominating mechanisms for N_2_O release into the atmosphere. For characterizing such scenarios, the development of sensor systems capable of *in-situ* in-field determination with suitable sensitivity and reliability are an essential demand. Conventionally, gas chromatography with electron capture detector (ECD) is applied during laboratory studies on collected environmental samples providing high sensitivity and accuracy^9^. However, extended sampling and measurement times, and the dimensions and operational requirements of the instruments render them less feasible for in-field scenarios, and certainly do not allow for potential usage as drone- or unmanned aerial vehicle (UAV)-mounted sensing systems.

Among the optical sensors, the mid-infrared spectral region (MIR; 2.5–25 μm) has been widely utilized for N_2_O detection^11,16,17^ utilizing IR-absorption phenomena^18^. To achieve the desired sensitivity, usually multi-pass gas cell providing extended absorption path lengths are applied. Again, despite many studies demonstrating suitable sensitivity the involved cost of instrumentation and optically sophisticated gas cells along with bulky system dimensions to date limit extended field applications of such MIR analyzer systems.

As a viable alternative to complex gas cells, hollow waveguides (HWG) serving as a light pipes made of dielectric materials or metals with a coaxial channel enabling radiation propagation by reflection at the inside wall have emerged^19^. If gaseous samples are injected into the hollow core, the HWG may simultaneously act as a miniaturized gas cell providing several meters of optical path length yet demanding only minute gas sample volumes (i.e., several milliliters). Simultaneously, HWGs serve as an optical waveguide propagating radiation at rather low attenuation losses. Consequently, HWGs facilitate the development of miniaturized MIR gas sensing systems providing extended and well-defined optical path lengths with high optical throughput, while maintaining fast response times (<1 min) due to the small transient gas sample volume. The utility and adaptability of HWGs coupled to FTIR spectrometers operating in the MIR spectral range for various gas sensing applications has extensively been described in literature^20–22^.

Substrate-integrated hollow waveguide (iHWGs) constitute a new generation of hollow waveguides pioneered by the research team of Mizaikoff and collaborators^23^ with exceptionally compact substrate dimensions (e.g., made from aluminum), an adaptable (i.e., designable) optical path lengths via the integration of meandered hollow waveguide channels at virtually any desired geometry into an otherwise planar substrate. Furthermore, a minute volume of gas sample is required for analysis (i.e., few hundreds of microliters), and the short transient residence time of these sample volumes within the active transducer region facilitate real-time monitoring rendering iHWGs tailorable to a wide range of application scenarios^24–27^.

Combining the iHWG technology with a compact preconcentrator device, we demonstrate in the present study the first field-deployable, compact MIR N_2_O gas sensor operating at the demanded concentration levels. Nitrous oxide was collected from gaseous samples using molecular sieve 5 A during 10 min at a gas sample flow rate of 200 mL min^−1^ corresponding to a total sampled volume of 2 L, and was then thermally desorbed at 270 °C for 1 min into a gas volume of 5 milliliters, thus facilitating an enrichment effect by a factor of 400. For quantitative analysis, the pronounced fundamental v_3_ MIR band of the N_2_O molecule occurring at 2222–2177 cm^−1^ range was evaluated enabling a limit of detection of 5 ppbv.

## Results

### Optimization of the preconcentration parameters

Several parameters were evaluated and optimized in order to maximize the analytical signal intensity during the preconcentration step^28^. N_2_O molecules were trapped using molecular sieve 5 A as sorbent, and were then thermally desorbed into nitrogen with the desorption flow carrying the molecules into the iHWG. Sampling of 5 ppmv of N_2_O at 200 mL min^−1^ for 10 min was used during the optimization. The desorption flow was evaluated in the range from 3 to 20 mL min^−1^ (see Figure S1) yielding an optimized gas flow of 5 mL min^−1^ used during all further experiments. The desorption temperature and time were optimized at 270 °C for 1 min yielding reliable and reproducible N_2_O-MIR signals (Figures S2 and S3). The improvement factor resulting from the introduction of the preconcentration step was calculated as approx. 500 by comparison of the integrated peak area associated with the N_2_O signal at 2222–2177 cm^−1^ of standard mixtures at 500 ppm directly injected to the detection system, and 2.5 ppm subjected to the preconcentration procedure. Figure S4 shows the spectrum at each condition.

### Analytical figures-of-merit

For quantification, a calibration function was established based on the evaluation of the peak area with integration boundaries from 2222 to 2177 cm^−1^ vs. the nitrous oxide concentration. For each concentration, the mean value of three independent replicate measurements was calculated. The resulting goodness of the fit of the linear calibration function was determined at r^2^ > 0.99 in the concentration range of 0.1 to 2.5 ppmv of nitrous oxide following A = 0.813 [N_2_O] + 0.0118. The calculated limit of detection (LOD) was considered at three-times the standard deviation of the blank signal, and was determined at 5 ppbv. The obtained analytical figures-of-merit are summarized in Table 1.

**Table 1:** Summary of analytical figures-of-merit obtained for the iHWG-FTIR nitrous oxide gas sensing system.

Parameter	Value
	N_2_O
Limit of detection (3*SD of blank)	5 ppbv
Correlation coefficient	0.9983
Linear range	0.1–2.5 ppmv
Regression equation	A = 0.813 [N_2_O] + 0.0118

### Evaluation of spiked and real-world samples

In order to evaluate the utility of the proposed methodology for analyzing real-world atmospheric samples, Tedlar^®^ gas sampling bags were (i) filled with untreated atmospheric air collected outdoors and quantitatively spiked with N_2_O obtaining a final concentration of 0.5 and 2.5 ppmv for calibration purposes, and (ii) filled only with untreated atmospheric air for analyzing the real-world background concentration based on the established calibration. Thereafter, these samples were inserted into the preconcentration system by suction using a pocket pump set at 200 mL min^−1^ for 10 min, and were then analyzed as previously described for the calibration samples. The obtained concentrations of the spiked samples derived from the calibration function were 0.42 ± 0.09 and 2.39 ± 0.11 ppmv resulting in recovery rates of 84% and 96%. The N_2_O concentration was then determined in untreated atmospheric samples was 306 ± 1 ppbv. This value is in excellent agreement with the anticipated atmospheric background concentration of N_2_O (i.e., on average 330 ppbv).

## Discussion

**Figure 1:**
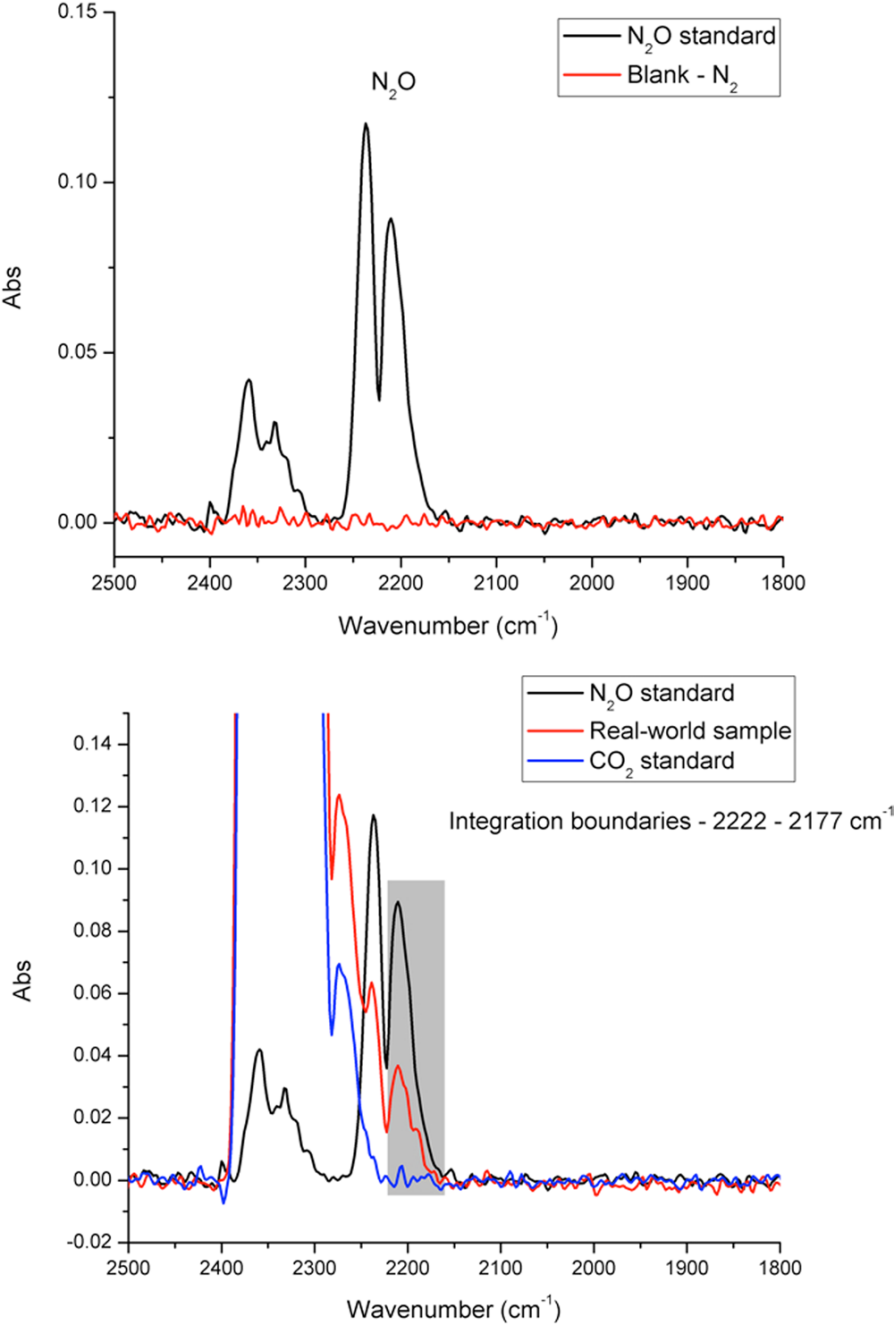
(**a**) IR spectra of N_2_O standard at 2.5 ppmv and blank obtained with optimized preconcentration parameters; **(b)** spectra of real-world sample, N_2_O, and CO_2_ with the integration boundaries used for quantification.

Nitrous oxide has a pronounced absorption band within the mid-infrared spectral range associated with the fundamental ν_3_ vibration of the N_2_O molecule located at 2270–2160 cm^−1^ (Fig. 1)^29^. However, a spectral interference caused by broadening of the CO_2_ band has been identified during real-world sample evaluation. This interference was reduced by limiting the integration boundaries associated with the N_2_O signal at 2222–2177 cm^−1^. Figure 1 exemplarily shows the spectrum of N_2_O, CO_2_, and the real-world sample highlighting the applied signal integration boundaries.

**Figure 2:**
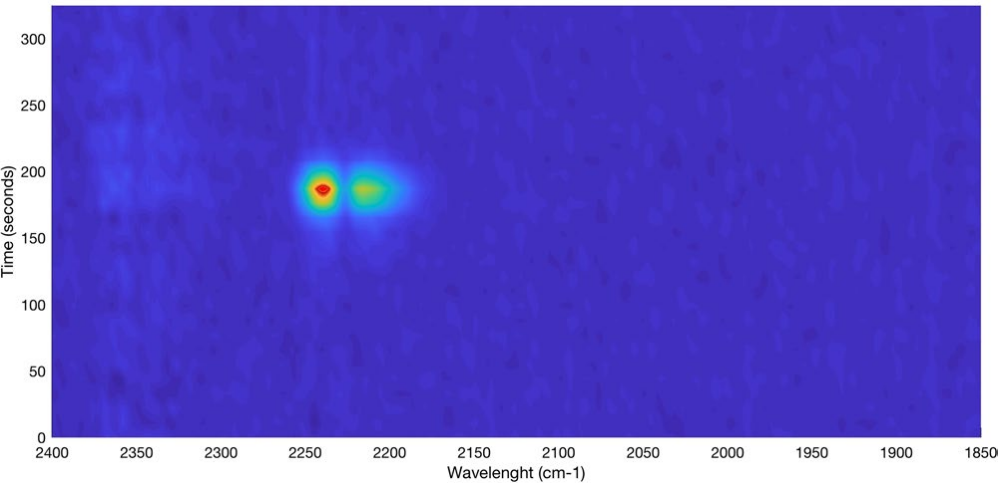
Contour plot (top view) of the temporal progression of the N_2_O IR signal during the desorption (blue – low concentration/IR-signal; red – high concentration/IR-signal).

The relation between the absorption signal and desorption time is demonstrated using nitrogen as desorption gas at a flow rate of 5 mL min^−1^. As evident in Fig. 2, no spectrum is recorded via the iHWG in the beginning of the desorption procedure, which is attributed to the dead volume between the iHWG-FTIR sensor and the preconcentrator. Approximately 150 s after sample desorption, the IR signal characteristic for N_2_O abruptly increased reaching a maximum value after approx. 175 s, which was then continuously decreasing again to zero (i.e., at approx. 200 s). The preconcentrator-iHWG system was then purged with nitrogen for 200 s to ensure complete removal of N_2_O from the sorbent, thereby avoiding any memory effects.

A gas phase preconcentration-based analytical method may be also evaluated by its efficiency in trapping molecules passing through the solid sorbent. Fundamentally, the trapping efficiency increases with the sample flow rate up to a maximum value, and then decreases. For the current configuration, the sample flow rate was evaluated in the range of 50–200 mL min^−1^. A gas sample flow of 200 mL min^−1^ for 10 min was determined providing an optimum signal; however, the flow rate may readily be adapted to other measurement scenarios and sampling requirements.

In summary, in the present study N_2_O molecules were trapped at room temperature using molecular sieve 5 A, and were then thermally desorbed at 270 °C into the iHWG-FTIR detection system using nitrogen at a flow rate of 5 mL min^−1^. For quantitative purposes, integration boundaries from 2222–2177 cm^−1^ were utilized to avoid spectral interference by CO_2_. Using a sampling flow rate of 200 mL min^−1^ for 10 min enabled a limit of detection of 5 ppbv. The sensitivity of the method may readily be improved by increasing the sampling flow rate and the analysis time, and by increasing the absorption path length provided by the iHWG. Furthermore, alternative (i.e., more efficient) sorbent materials may further improve the achieved preconcentration effect. Smartly optimizing these parameters, it is immediately evident that the developed iHWG MIR sensor system provides a generic transducer platform that may be flexibly and modularly adapted to a wide range of monitoring scenarios. Besides, replacing the FTIR spectrometer with a suitable tunable quantum cascade laser (tQCL) light source should result in a significant reduction of the physical dimensions of the developed IR sensing device.

## Methods

### Materials and FTIR spectrometer

Nitrous oxide and nitrogen were acquired from MTI Industriegase AG (Neu-Ulm, Germany). The sample preconcentration tube was custom-made from a quartz tube in U-shape with an inner diameter of 3.5 mm. The effective packaging length containing molecular sieve 5 A (Sigma-Aldrich, St Louis, USA) – a crystalline metal aluminosilicate frequently applied as solid sorbent for gaseous compounds^30^ – was 40 mm. Thermal desorption was performed using a heating wire wrapped around the tube and connected to a voltage/current controller (Basetech, BT-305, Germany). All measurements were performed using a compact portable FTIR spectrometer (Alpha OEM, Bruker Optics Inc, Ettlingen, Germany) equipped with a liquid nitrogen cooled mercury-cadmium-telluride (MCT) detector (FTIR-22.1.00, Infrared Associates, Stuart/FL, USA). A straight-line iHWG made from polished aluminum providing an integrated hollow waveguide channel (i.e., absorption path length) of 15 cm at device dimensions of 150 × 46 × 17 mm (length x width x depth) was coupled to the spectrometer using gold-coated off-axis parabolic mirrors with a focal length of 1′′ (Thorlabs, Dachau, Germany). IR spectra were recorded in the range of 4000–650 cm^−1^ at a spectral resolution of 4 cm^−1^; 10 spectra were averaged per measurement. The OPUS 7.2 software package (Bruker Optics Inc, Ettlingen, Germany) was used for data acquisition and evaluation.

### Measurement protocol

**Figure 3:**
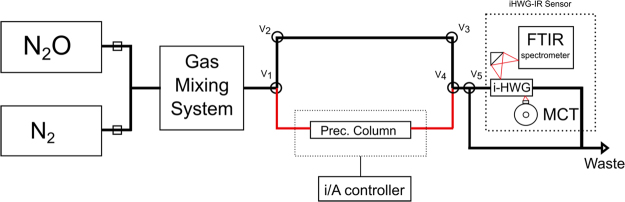
Scheme of the N_2_O sample generation system coupled to the iHWG-FTIR sensor and preconcentrator.

The experimental setup is schematically shown in Fig. 3. Polytetrafluoroethylene tubes with Luer-lock^®^ adapters were used to connect the system components. Three-way gas valves were adjusted for switching different sample pathways during a measurement routine. The calibration protocol was executed in four steps (a–d) as follows:

- **Standard gas mixtures:** Nitrous oxide and nitrogen cylinders were used to prepare standard gas mixtures. Suitable gas flow rates of N_2_O and N_2_ were mixed and delivered to the sensing system at a flow rate of 200 mL min^−1^. The sample preparation was carried out for a period of 3 min to obtain homogenous solutions. Then, valves V_1_ and V_4_ were set to enable the passage of the sample through the preconcentration tube.
- **Sampling:** Samples were introduced into the preconcentration tube containing the sorbent at a gas flow rate of 200 mL min^−1^ for 10 min (i.e., resulting in sampling 2 L of each sample). For the standard gas mixtures, the flow was regulated using the gas mixing system. For real-world samples, Tedlar® gas sampling bags containing air samples were attached to the preconcentration tube, and the gas sample was transported through the system by suction using a pocket pump (Model 222-3, SKC, Dorset, United Kingdon) with a calibrated gas flow rate of 200 mL min^−1^.
- **Cleaning:** After sampling, the valves V_1_ and V_4_ were set to close the preconcentration pathway. Nitrogen at a flow rate of 200 mL min^−1^ was used to regenerate the system for 3 min. Thereafter, a background IR spectrum was collected averaging 100 scans.
- **Thermal desorption/Data acquisition:** After cleaning, the preconcentration tube was heated (i.e., via the resistive heating wire controlled by a temperature-calibrated voltage/current controller @ 5.3 A and 18.5 V) for 1 min. For the elution procedure, nitrogen was inserted into the preconcentration tube via V_1_ and V_4_ at a flow rate of 5 mL min^−1^, thereby carrying the desorbed N_2_O molecules to the iHWG-FTIR sensing device through valve V_5_.

## Electronic supplementary material

Supplementary Information
