# Supplementary material for: A Hyphenated Preconcentrator-Infrared-Hollow-Waveguide Sensor System for N2O Sensing
Source: Sci Rep. 2018 Apr 12;8:5909. doi: 10.1038/s41598-018-23961-8 (PMC5897552; doi:10.1038/s41598-018-23961-8)
Supplement: Supplementary file 1 — Supplementary Information [file 41598_2018_23961_MOESM1_ESM.pdf]

SUPPLEMENTARY INFORMATION

A HYPHENATED PRECONCENTRATOR-INFRARED-HOLLOW-WAVEGUIDE SENSOR SYSTEM FOR N_2O SENSING

João Flavio da Silveira Petruci[1,2], Andreas Wilk[2], Arnaldo Alves Cardoso[1], and Boris Mizaikoff[2]*

[1] São Paulo State University, Department of Analytical Chemistry, UNESP, CEP 14800-970, Araraquara, SP, Brazil

[2] Ulm University, Institute of Analytical and Bioanalytical Chemistry, 89081, Ulm, Germany

*boris.mizaikoff@uni-ulm.de

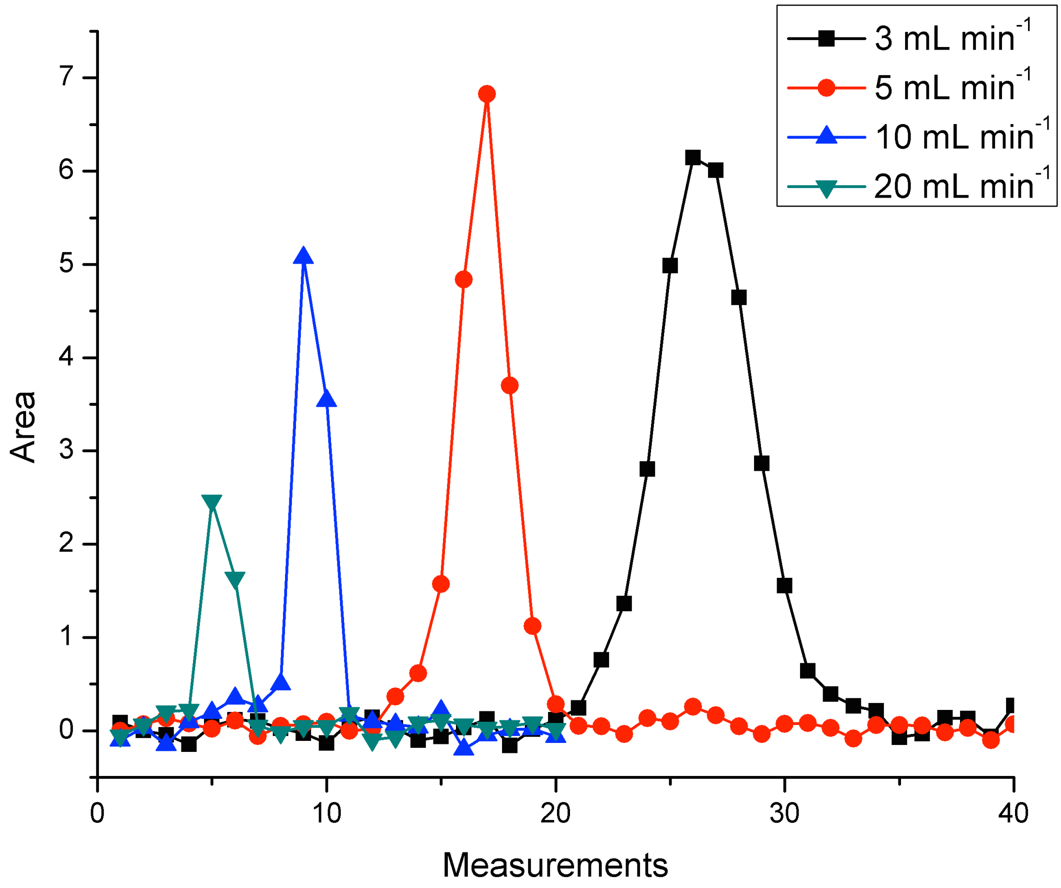

Figure S-1. Evaluation of desorption flow of a sample preconcentration of 5 ppmv of N_2O.

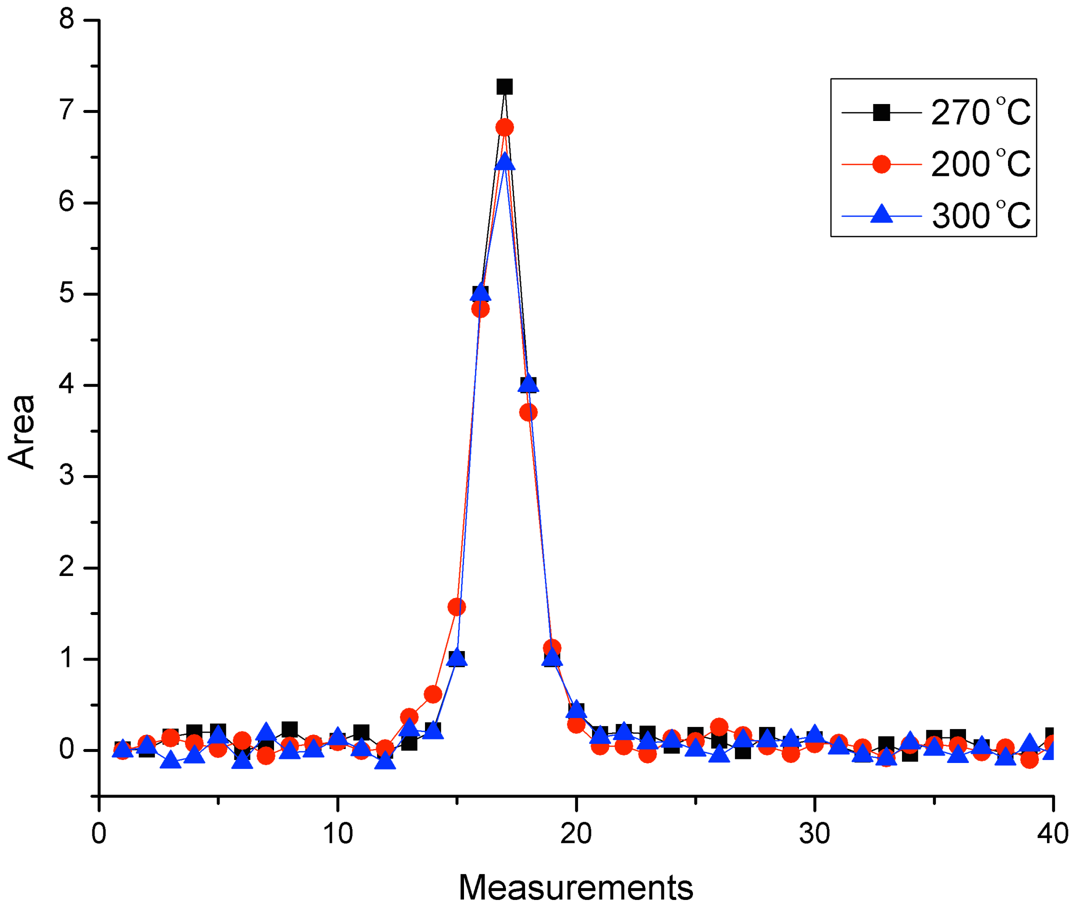

Figure S-2 Evaluation of desorption temperature of a sample preconcentration of 5 ppmv of $N2O$.

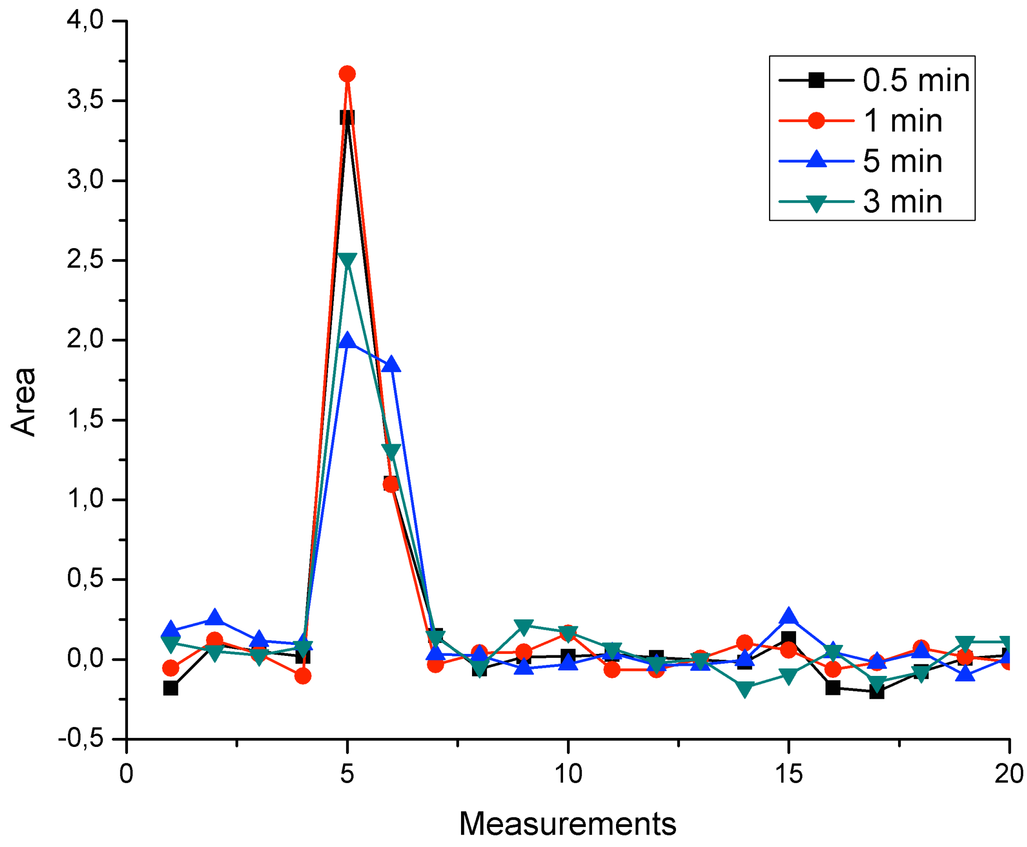

Figure S-3. Evaluation of heating time of a sample preconcentration of 5 ppm of N_2O.

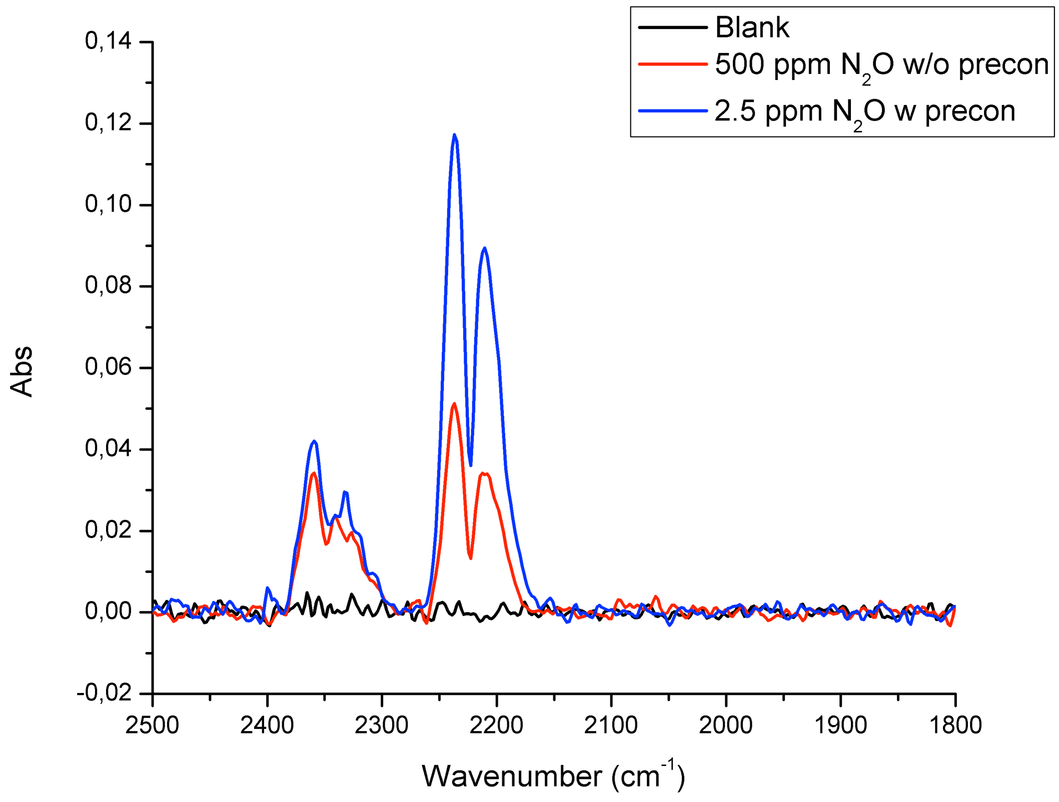

Figure S-4. Comparison of the analytical signal of 2.5 ppm of N2O with (blue line) and 500 ppm of N2O without (red line) preconcentration.
